# Syntheses of Gold and Silver Nanocomposite Contact Lenses via Chemical Volumetric Modulation of Hydrogels

**DOI:** 10.1021/acsbiomaterials.2c00174

**Published:** 2022-04-25

**Authors:** Ahmed E. Salih, Mohamed Elsherif, Fahad Alam, Bader Alqattan, Ali K. Yetisen, Haider Butt

**Affiliations:** † Department of Mechanical Engineering, Khalifa University, P.O. Box, Abu Dhabi 127788, United Arab Emirates; ‡ Department of Chemical Engineering, Imperial College London, London SW7 2AZ, U.K.

**Keywords:** biomaterials, nanocomposites, nanoparticles, blue light, wearables, contact lenses

## Abstract

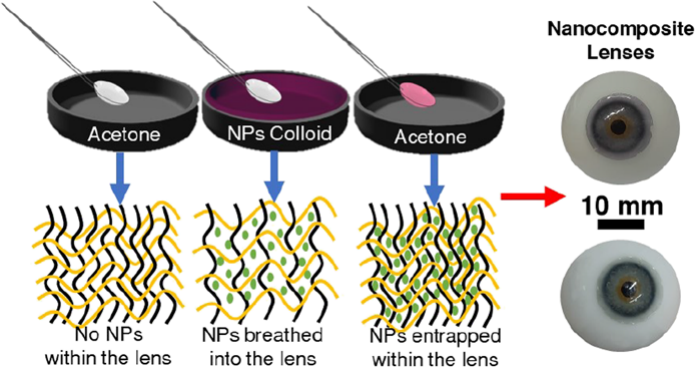

Integration of nanomaterials into hydrogels has emerged as a prominent research tool utilized in applications such as sensing, cancer therapy, and bone tissue engineering. Wearable contact lenses functionalized with nanoparticles have been exploited in therapeutics and targeted therapy. Here, we report the fabrication of gold and silver nanocomposite commercial contact lenses using a breathing-in/breathing-out (BI–BO) method, whereby a hydrated contact lens is shrunk in an aprotic solvent and then allowed to swell in an aqueous solution containing nanoparticles. The morphology and optical properties of the gold and silver nanoparticles were characterized through transmission electron microscopy and ultraviolet–visible spectroscopy. The transmission spectra of nanocomposite contact lenses indicated that the nanoparticles’ loading amount within the lens depended primarily on the number of BI–BO cycles. Nanocomposites were stable for a minimum period of 1 month, and no nanoparticle leaching was observed. Wettability and water content analysis of the nanocomposites revealed that the contact lenses retained their intrinsic material properties after the fabrication process. The dispersion of the nanoparticles within the contact lens media was determined through scanning electron microscopy imaging. The nanocomposite lenses can be deployed in color filtering and antibacterial applications. In fact, the silver nanocomposite contact lens showed blue-light blocking capabilities by filtering a harmful high-energy blue-light range (400–450 nm) while transmitting the visible light beyond 470 nm, which facilitates enhanced night vision and color distinction. The ease of fabricating these nanocomposite contact lenses via the BI–BO method could enable the incorporation of nanoparticles with diverse morphologies into contact lenses for various biomedical applications.

## 1 Introduction

Recent interest and need for continuous real-time health monitoring have increased and facilitated the progress of noninvasive wearable techniques and devices, yet early methods were primarily aimed at monitoring physical activities like the step count and heart rate.^1^ Wearables, such as skin patches, smart contact lenses, and wrist straps, enabled researchers to target and real-time monitor major diseases and conditions including diabetes, glaucoma, and cystic fibrosis.^1−7^ Currently, smart wearables are not only being used in patients’ health monitoring but also in combating and correcting ocular diseases and deficiencies like blindness, color vision, and dyslexia.^8−10^ Of these smart wearables, contact lenses have been the most prevalent, owing to their flexible nature and ease of use, along with simplicity of adopting noninvasive sensing techniques onto them.^11−15^ The latter paved the way for the development of contact lenses for monitoring various physiological attributes, such as tear glucose concentration,^6^ corneal temperature,^16^ and ocular pH.^7^

Moreover, integration of nanomaterials, such as silver (Ag) and gold (Au) nanoparticles, into contact lenses has emerged as a prominent tool for the development of smart contact lenses. Owing to their surface plasmon resonance (SPR), Au and Ag nanoparticles are highly efficient in absorbing and scattering specific portions of the visible light electromagnetic spectrum, which occurs as a result of the excitation of the conduction electrons on the metal’s surface at specific finite wavelengths.^17−20^ Au and Ag nanoparticles have been incorporated into contact lenses and their unique SPR exploited for a wide range of biomedical applications. For instance, gold nanoparticles were recently added into contact lenses in an attempt to aid colorblind patients.^21^ The reported gold nanocomposite contact lenses (NCCLs) were effective in absorbing undistinguishable colors of the patient’s spectrum and were similar in optical performance to commercial colorblind glasses. Authors reported that the addition of the gold nanoparticles did not adversely affect the material properties of the contact lenses, specifically their contact angle and water content.^21^ Using a similar methodology, the same group fabricated silver nanoparticle-loaded contact lenses to enhance color distinction in blue-yellow color vision deficient patients; the reported physiochemical properties of the lenses were analogous to the ones previously synthesized.^22^ Liu et al. also incorporated gold nanoparticles into polyvinyl alcohol (PVA) contact lenses as a preventive measure against accidental exposure to green laser.^23^ Nanoparticles were well dispersed within the lens, and the nanocomposite contact lens showed excellent laser protection efficacy. Authors also reported that their lenses generate heat when exposed to laser, and this heat can be potentially applied for meibomian gland dysfunction (MGD) dry eye treatment since the resulting temperature is higher than the melting temperature of the meibomian lipids that are critical for treating MGD dry eyes. Further, Kharaghani et al. added silver and copper nanoparticles into PVA contact lenses prior to polymerization.^24^ They tested the antibacterial properties and cytotoxicity of these lenses; lenses, having both copper and silver–copper nanoparticles, were not cytotoxic. Yet, silver nanoparticles embedded within PVA contact lenses were found to be toxic.^24^

All of the aforementioned nanocomposite lenses were developed based on in situ synthesized contact lenses in which nanoparticles are mixed with the contact lens monomer solution prior to polymerization and, thus, formation of the contact lenses in their gel form. One of the main concerns in the synthesis of such nanocomposite lenses is not knowing the physiochemical properties of the fabricated contact lenses, such as the modulus, wettability, and oxygen permeability, which are difficult to characterize and require sophisticated equipment. However, those properties are well established and documented for commercial contact lenses. Hence, the lack of such lenses in the literature necessitates the development of a methodology for doping commercial contact lenses with gold and silver nanoparticles.

Dipping or soaking commercial contact lenses in colloidal nanoparticles usually does not result in permeation of the NPs through the lenses’ pores. Hence, the unique breathing-in/breathing-out (BI–BO) method of incorporating nanoparticles into gels is utilized.^25−27^ Introduction of nanoparticles into the gel occurs in two steps: the swollen gel is placed in an aprotic solvent and gets deswollen by expelling water (breathing-out); the second step involves dipping the shrunken gel into an aqueous solution containing nanoparticles (breathing-in). Upon the next BO step, nanoparticles are preserved within the gel’s matrix. The aforementioned method has been reportedly used for few nonionic polymeric gels such as polyacrylamide.^26−28^ Nonetheless, this is the first study reporting the use of this method for the incorporation of nanoparticles into commercial contact lenses. Acuvue Trueye (narafilcon A), a common commercial contact lens, was utilized in this study. Ease of loading gold and silver nanoparticles into commercial contact lenses can make these nanocomposites suitable for various applications, one of which is color filtering. Indeed, after evaluating the lenses’ properties and determining whether the process adversely affects them, we evaluated their potential as smart wearables, particularly as antibacterial, color vision deficiency, and blue-light filtering contact lenses. Since silver nanoparticles absorb visible light in the range of 400–490 nm (depending on their respective size), the filtering efficacy of the silver nanocomposite contact lens developed in this study was compared to that of commercial blue-light filtering wearables.

Prior to that, the morphology of the parent nanoparticles embedded within these lenses was studied through transmission electron microscopy (TEM), and their transmission spectra were recorded through ultraviolet–visible (UV–vis) spectroscopy. The nanocomposite contact lenses were, then, characterized for their optical and material properties, and the effect of varying the BI–BO cycles on them was assessed. Scanning electron microscopy (SEM) was finally used to examine the distribution of the NPs within the contact lenses.

## 2 Experimental Section

### 2.1 Chemicals

Gold nanoparticles (20 nm), stabilized in phosphate buffer solution (PBS) with 0.1 mM concentration, and 40 nm silver nanoparticles, dispersed in PBS with a concentration of 0.02 mg/mL, were purchased and used from Sigma Aldrich as is without further purification. Acetone, as an aprotic solvent, was also purchased from Sigma Aldrich. 1-Day Acuvue TruEye contact lenses were obtained from Acuvue, Johnson & Johnson.

### 2.2 Fabrication

**Figure 1:**
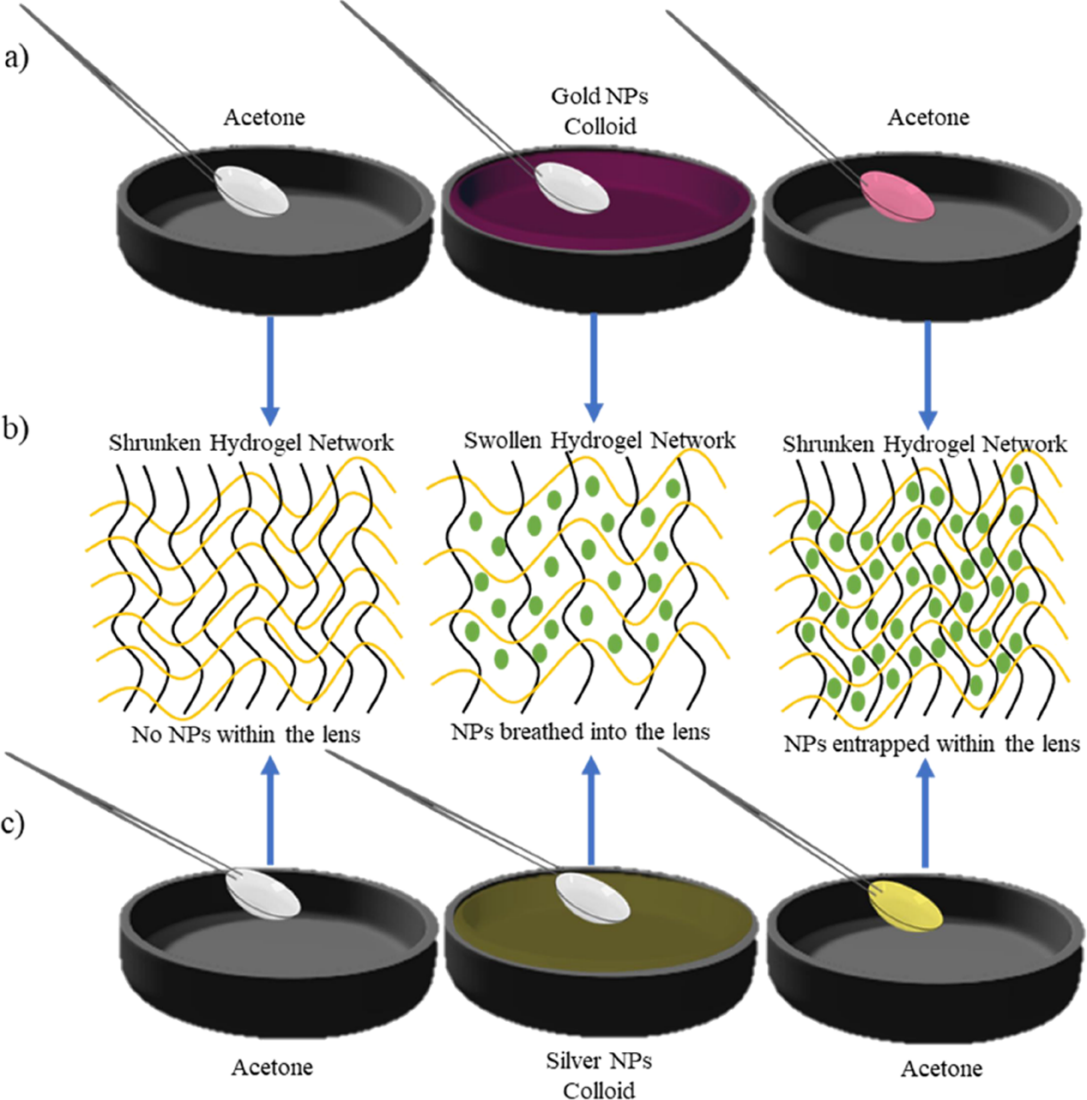
Fabrication of nanocomposite contact lenses. (a,c) Schematics of the BI–BO fabrication process. (b) Physical entrapment of the nanoparticles within the hydrogel using alternating cycles of deswelling in an aprotic solvent (acetone) and swelling in colloidal nanoparticles.

The incorporation of the nanoparticles into the lenses was done through the unique BI–BO method (Figure 1). The Acuvue contact lenses were placed in 10 mL of acetone for 2 min and then placed in 10 mL of colloidal nanoparticles. This cycle was repeated multiple times for both sets of nanoparticles to show its effect on the optical and material properties. The contact lenses were then washed using deionized water to remove any unabsorbed nanoparticles. Samples were kept for further analysis in a contact lens storage solution at 25 °C.

### 2.3 Characterization

Primary characterizations were done to assess the optical and material properties of the NCCLs, yet the optical and morphological properties of the nanoparticles, themselves, were studied prior to the lens characterization. The transmission spectrum of the colloidal nanoparticles was obtained using USB 4000+, a UV–vis spectrophotometer, which was connected to an optical microscope. The utilized spectrophotometer operates in the range of 200–1100 nm. Furthermore, 500 μL was deposited into a cuvette, and the transmission spectrum was recorded using OceanView software. The morphology of the nanoparticles in their solutions was obtained utilizing a Tecnai TEM 200 kV, which has a resolution of 0.24 nm, and voltage varying from 20 to 200 kV. Further, 15 μL of the NP solution was placed on copper mesh grids, purchased from TED PELLA, and allowed to dry at room temperature; this was repeated multiple times to have a considerable amount of NPs in the mesh grid. Size and distribution analysis of the resulting images was done using ImageJ software. Similarly, the transmission spectra of the NCCLs were obtained using the same procedure previously outlined. The latter was recorded at multiple BI–BO cycles, namely, 5, 10, 15, and 20, to show their subsequent influence on the optical properties of the contact lenses. The stability of the nanoparticles within the lenses was also assessed by measuring their transmission spectra over a month.

Furthermore, the wettability and water retention of the contact lenses were evaluated. For the wettability analysis, the static contact angle was measured using a sessile drop method where a stored and hydrated contact lens was placed on a glass slide in the contact angle setup (Figure S1). Then, 3 and 5 μL of water droplets were placed on the surface of each sample, and five images of the water drops at different spots within the sample were captured. The contact angle was then obtained using the “LBADSA” external plugin for measuring sessile drops in ImageJ software (Figure S2). Likewise, the water retention of the nanocomposite contact lenses was determined by recording their dry weight and comparing it with their fully hydrated weight. To ensure that the samples were completely dried, they were placed in a vacuum oven at 50 °C for 2 h. The samples were then immersed in deionized water for 72 h to ensure the maximum water retention possible. Their total weight and corresponding water content were analyzed at multiple time intervals to assess the transient water retention using the equation below:
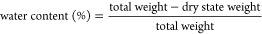
1

The characterizations were performed on one clear (undoped) contact lens, a lens treated with five BI–BO cycles without NPs (only water), and two doped nanocomposite lenses to compare their material and optical properties and assess the efficacy of this novel method. Furthermore, FEI Nova NanoSEM 650, which has an electron beam resolution of 0.8 nm, was used to study the morphology of the nanoparticles within the NCCL. For the SEM imaging, the contact lens was sheared to examine the cross-section of the sample and was then coated with a 7.5 nm layer of platinum, to avoid the charge-up effect.

## 3 Results and Discussion

**Figure 2:**
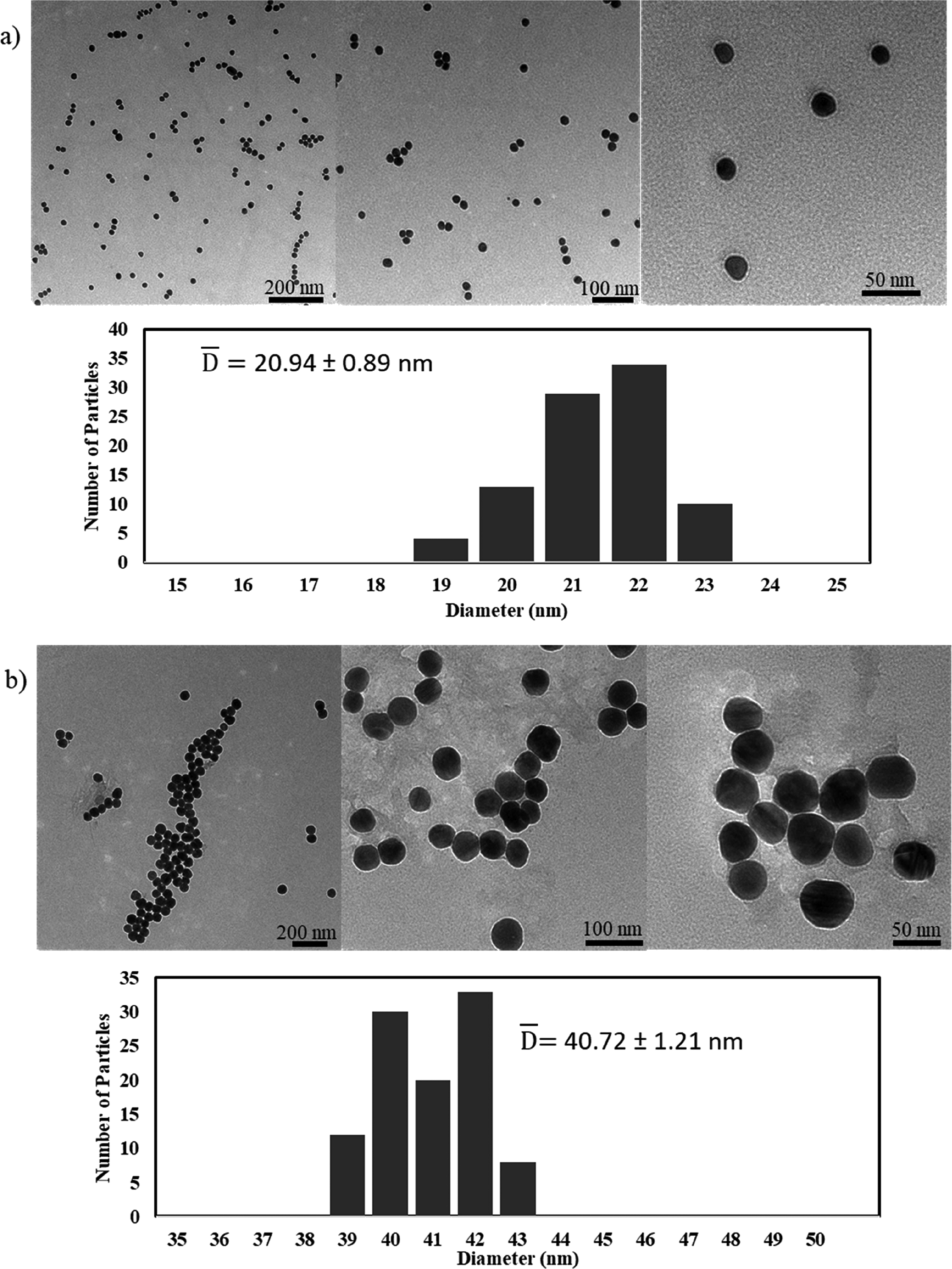
Morphology characterization of the nanoparticles. (a) 20 nm gold nanoparticles. (b) 40 nm silver nanoparticles. Top: TEM images at different magnifications. Bottom: Size distribution histograms

The size and distribution of the nanoparticles were obtained through TEM images and are shown in Figure 2. For each set of nanoparticles, a minimum of 100 particles were imaged and analyzed to get an accurate size distribution of the nanoparticles. As illustrated from the histograms, the average diameters of the gold and silver nanoparticles were 20.94 ± 0.89 and 40.72 ± 1.21 nm, respectively. Standard deviations of both sets of nanoparticles were less than 5%, indicating their size homogeneity. As for the distribution of the nanoparticles, TEM images show that gold nanoparticles were, overall, more evenly dispersed (Figure 2a) as compared to the silver nanoparticles, which showed more aggregates and formed clusters (Figure 2b). It is worth noting that while preparing the samples for TEM imaging, the concentration and generally the chemical composition of the nanoparticle solution were not altered. Hence, these were the morphologies of the nanoparticles utilized in the contact lenses.

**Figure 3:**
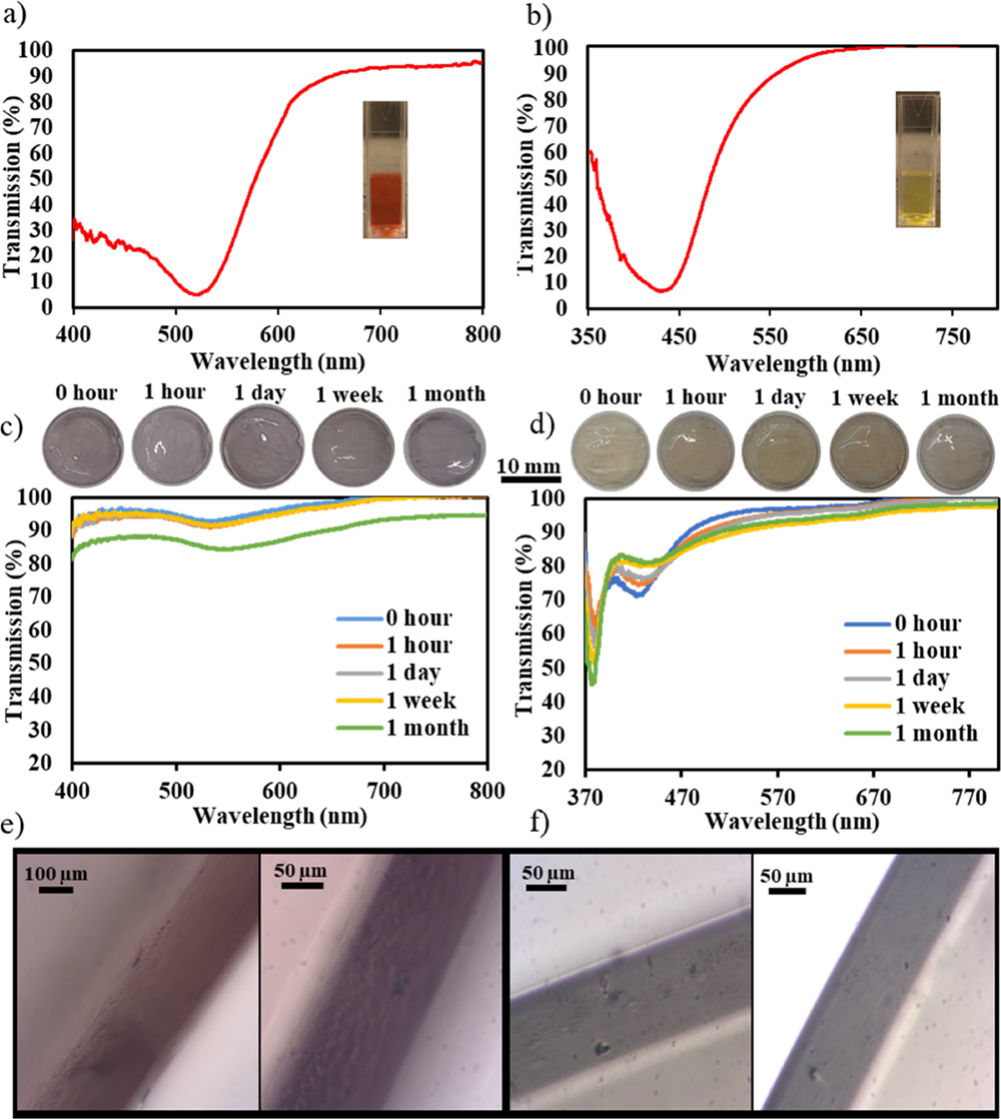
Transmission spectrum of the nanoparticles in their solution. (a) 20 nm gold nanoparticles. (b) 40 nm silver nanoparticles. The inset shows images of the nanoparticle solutions. Transmission spectra of the (c) gold and (d) silver nanocomposite lenses, developed using the breathing-in/breathing-out method, over a one-month period with their images at each time interval. Microscopic images (transmission mode) of the (e) gold and (f) silver nanocomposite contact lens cross-section at different magnifications showing a homogeneous color.

Moreover, the transmission spectrum of each nanoparticle solution was measured and is demonstrated in Figure 3a,b. The surface plasmon resonance or transmission dip of the gold and silver nanoparticles occurred at 522 and 430 nm, respectively, and both NPs blocked more than 90% at these wavelengths. Moreover, the full width at half maximum (FWHM), which quantifies the bandwidth of the transmission dip, was 43 and 93 nm, respectively. The discrepancy in FWHM between both nanoparticles could be attributed to their degree of dispersion, shown through the TEM images. The FWHM of the transmission dip is adversely affected by nanoparticle aggregation, which was evident from the wide transmission dip in the spectra of the aggregated silver nanoparticles. Aggregation causes the conduction electrons of one nanoparticle (responsible for SPR) that is close to another one to be delocalized and shared with it, which decreases the energy and frequency required to resonate them; this translates to either a slight broadening of the transmission dip or, in cases of complete aggregation, to disappearance of the SPR. In addition, silver nanoparticles generally exhibit larger bandwidths at the transmission dip compared to gold nanoparticles, which is well explained by the Mie scattering theory.

The lenses were then doped with the nanoparticles using the BI–BO method described earlier, and the transmission spectra of both gold and silver NCCLs after 15 BI–BO cycles were measured. It is vital to assess the leakage or diffusion of the nanoparticles from the lenses; this was done over a one-month period, and the resulting spectra are demonstrated in Figure 3c,d. The initial transmission spectra (at 0 h) of the gold and silver NCCLs showed a transmission dip at 532 and 433 nm, respectively. It is worth noting that the transmission dip at 380 nm of the silver NCCL is an inherent UV filtering feature of the Acuvue TruEye contact lens. Comparing the SPR of the nanocomposite lenses to the parent nanoparticles, one can note a slight shift, which is due to the change in the refractive index of the medium. The refractive index of narafilcon A is 1.41 as reported by Acuvue, whereas the refractive index of the NP solution is that of water (1.33). The change of SPR wavelength with the refractive index was explained thoroughly be Salih et al.^21^ It occurs mainly due to two reasons: the first is the consequent change of the light wavelength at the nanoparticles’ vicinity resulting from the medium’s refractive index variation, and the second occurs due to the polarization of the nanoparticle dielectric medium, which causes a variation of the effective charge on the NPs’ surface. This variation is resembled by a change in the SPR wavelength in the optical spectra.

In contrast to the silver nanocomposite lens, the transmission bandwidth of the gold nanocomposite lens was wider, and the NPs’ sharp dip shown in Figure 3a flattened out. Also comparing the color of the gold NP solution, which was reddish, to the color of the NPs in the NCCL (Figure 3c), one can note that the transfer of the nanoparticles to the contact lens material (narafilcon A) might have caused this shift in color. Indeed, the latter indicates that the gold nanoparticles aggregated; however, their aggregation was not caused by the BI–BO method since similar color changes were not observed in the silver nanoparticles after adding them to the lens. A probable cause for the aggregation and, consequently, the wide transmission dip of the gold NCCL is the size of the NPs relative to the silver NPs. In fact, nanoparticles smaller than 20 nm have a higher surface to bulk volume ratio, and hence, they have higher surface energies as compared to relatively bigger particles (>40 nm). Therefore, when changing their medium (from water to the polymeric contact lens matrix), aggregation of most gold nanoparticles, in order to reduce their overall free energy, caused the apparent abrupt change in their transmission spectra from their colloidal state. Variations in the aggregation state of NPs, due to size discrepancies, were also observed in a previous study that utilized silica nanofillers with polyamide 6.^29^ Authors reported that excessive aggregation occurred when they mixed 12 nm nanofillers with their polymers, whereas 50 nm nanofillers were relatively more well dispersed when incorporated into the polymeric matrix.^29^ Another review study reported that the latter is mostly due to differences in the surface energy of the nanoparticles.^30^

Moreover, over the period of 1 month, the stability of both nanoparticles within the contact lenses was apparent as their transmission spectra remained unchanged, indicating the absence of NP leakage. Also, the fluid in which the NPs (tear fluid solution) were immersed was monitored by measuring its absorption/transmission spectra over a one-month period (Figure S6), which confirmed no NP leaching occurred. The stability of the nanoparticles within the matrix can be attributed to two reasons. First, gold and silver nanoparticles were capped with an agent (citrate/phosphate), which could have aided in the formation of hydrogen bonding between them and the polymer. The NPs could have also been physically entangled or attached to the contact lens. These observations were also noted in previous studies;^27,28^ hence, the utilization of the BI–BO method successfully incorporated nanoparticles into contact lenses, with minimal or no leakage observed. Cross-sectional microscopic images of both NCCLs at different magnifications were done and are shown in Figure 3e,f. The images were indicative of the homogeneity of the color within the lens and consequently the distribution of the nanoparticles, yet cross-sectional SEM images of the NCCLs were needed to confirm the latter.

**Figure 4:**
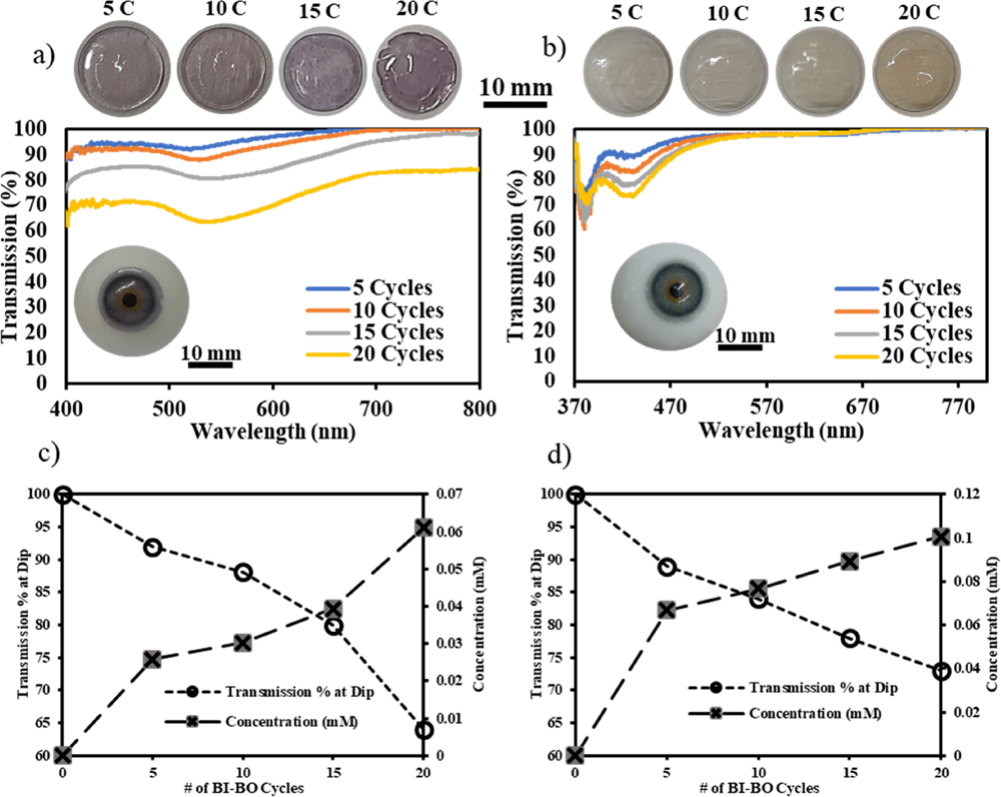
Transmission spectra of the (a) gold and (b) silver nanocomposite contact lenses at different breathing-in/breathing-out cycles with images at each cycle shown above the plot. The inset shows the contact lenses, after 20 BI–BO cycles, on an eye model. Effect of varying the number of BI–BO cycles on the transmission percentage at the dip and the NP concentration of the (c) gold and (d) silver nanocomposite contact lenses.

Furthermore, various applications, which utilize nanoparticles for their superior physical and chemical properties in contact lenses such as targeted therapy and many optical processes, require variant concentrations of nanoparticles within the lenses. Hence, this was demonstrated by altering the number of BI–BO cycles and examining the resulting effect on the optical spectra; transmission curves, at a five-cycle interval, were recorded and are shown in Figure 4. Both lenses were homogeneous in their color, which is evident from the eye models in Figure 4a,b and the microscopic images in Figure 3e,f, indicating that nanoparticles did not cluster in one specific area. For the gold NCCL shown in Figure 4a, the transmission dip occurred at 532 nm, and over a range of 20 cycles, the transmission at the dip decreased by 36% where it can be seen that the variation in transmission percentage was not consistent throughout the BI–BO cycle incrementation. Nonetheless, for the silver NCCL shown in Figure 4b, the SPR occurred at 433 nm, and up to 28% was blocked at the aforementioned wavelength. It is worth mentioning that in this test, the reference was an untreated (undoped) contact lens; hence, the transmission dip, initially at 380 nm, was not as clearly visible as the one in Figure 3d.

In contrast to the gold NCCL, variations in the BI–BO cycles of the silver NCCL yielded a more constant change in its transmission percentage at the dip (Figure 4d). Thomas et al. argued that the increase of the NP concentration within the polymer with the BI–BO cycles indicates that the nanoparticles entrapped from the previous cycle remain within the gel after each new breathing-out cycle. Indeed, placing the gel in an aprotic medium (acetone) after it was swollen in an aqueous solvent, containing nanoparticles, will not cause it to expel the newly introduced NPs, as the NPs are bonded with electron-rich nitrogen and oxygen species of the polymeric matrix.^26^ Therefore, each breathing-in part of the cycle introduces new nanoparticles into the contact lens; consequently, the concentration of the NPs within the gel is varied. Furthermore, one can note the high number of cycles undergone in this study as compared to the previous studies, which was primarily due to the low concentration of nanoparticles utilized (0.02 mg/mL); however, the concept of introducing the nanoparticles into the gel remains the same. It is also worth stating that few alternative contact lens materials to narafilcon A, such as senofilcon A and etafilcon A, were placed directly in both gold and silver nanoparticle solutions, yet no absorption occurred. Hence, it is the actual BI–BO cycle, which entraps the nanoparticles in the polymeric matrix and not just a simple NP diffusion. Similar observations were previously made with polyacrylamide.^28^

Moreover, the size of the NPs was important in their successful incorporation into the lenses and formation of the NCCLs. Prior to incorporating the current nanoparticles within the lens through the breathing method, attempts using silver and gold nanoparticles with sizes above 40 nm, in particular, 60, 80, and 100 nm, were made. These nanoparticles were also purchased from Sigma Aldrich and had the same concentration as the ones utilized in this study; nonetheless, their transmission spectra remained unchanged even after 30 BI–BO cycles, which indicates that the NPs were not able to penetrate into the gel’s matrix. Previous studies have shown similar observations; in fact, the largest size of NPs in all previous studies that utilized BI–BO for different hydrogels was 40 nm.^25−27^ In this regard, it is also worth noting that the pore size of the polymer’s matrix might have an important role in allowing the entrapment of the nanoparticles and their aggregates within the matrix. Evidently, largely sized nanoparticles form aggregates, which cannot permeate as smoothly through the gel’s matrix as the clusters formed by smaller nanoparticles can; hence, fewer of the largely sized nanoparticles would be entrapped within the gel’s matrix as compared to the small sized nanoparticles.

**Figure 5:**
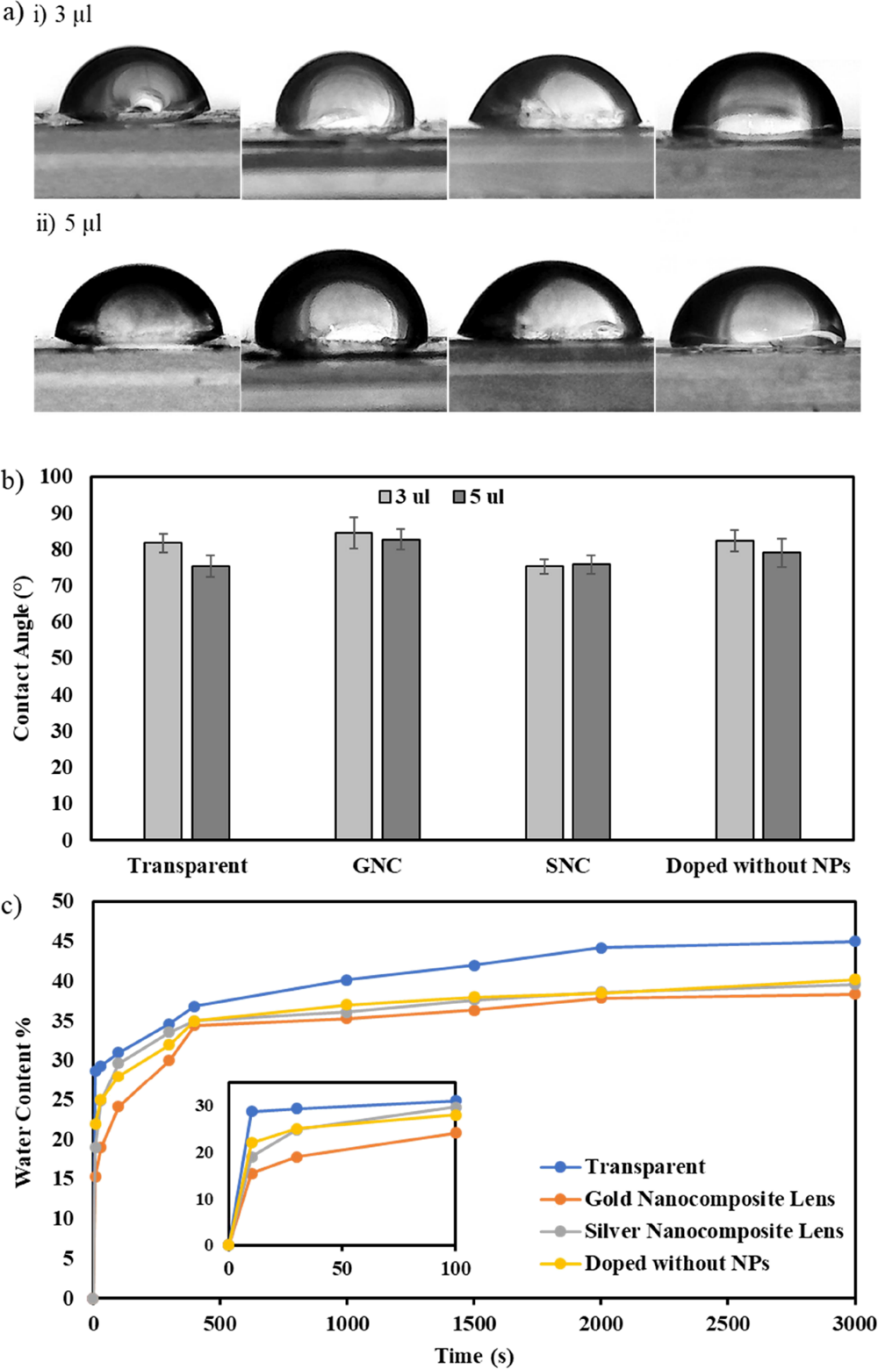
Material properties of the nanocomposite contact lenses: (a) Images of the contact angle measurements of undoped, gold, and silver nanocomposite lenses along with a lens that underwent five BI–BO cycles without using NPs (left to right) using the sessile drop method with (i) 3 μL and (ii) 5 μL water droplets. (b) Comparison between the contact angle measurements (five per sample) of the nanocomposite contact lenses, the transparent (untreated) lens, and the lens doped without using NPs, at 3 and 5 μL water droplets. (c) Water retention of the nanocomposite contact lenses compared to the untreated and doped without nanoparticles lenses at multiple time intervals. The inset shows the transient change of the water content of the four lenses.

To ensure that the BI–BO technique was not detrimental in altering the contact lens material properties, two of the most prominent CL properties, the contact angle and the water content, were measured and compared to those of an untreated sample along with a sample that underwent five BI–BO cycles without incorporating NPs. Figure 5a shows contact angle images, utilizing the sessile drop method, of the four samples (two fabricated NCCLs, untreated contact lens, and a contact lens treated with the BI–BO method without NPs) at two distinct volumes of water drops. Five trials per each sample and volume of droplet were recorded and averaged to minimize possible errors. Moreover, as shown in Figure 5b, the contact angles of the unaltered, doped without NPs, gold, and silver nanocomposite contact lenses at 3 μL were 81.8°, 82.5°, 84.6°, and 75.3°, respectively, while at 5 μL, the same set of samples had average contact angles of 75.4°, 79.2°, 82.8°, and 75.9°, respectively. Clearly, differences among the four tested samples were very insignificant to suggest any specific trend. The samples were generally hydrophilic; however, their contact angles were higher than the average contact angle of HEMA hydrogels, which is in the range of 60–70°. Copolymerization of HEMA and wetting agents like polyvinyl-pyrrolidone (PVP) with hydrophobic silicone-based hydrogels could have caused this decrease in surface wettability.^31^ Further, standard deviations were all less than 5%, which indicates that the measurements were close to each other with minimal errors. Also, no noticeable differences were observed when increasing the droplet volume from 3 to 5 μL. Therefore, it can be asserted that the incorporation of the nanoparticles into the contact lenses through the BI–BO method did not alter the inherent surface chemistry and wettability of the samples.

The second measured property of the contact lenses was their water retention, and the results are shown in Figure 5c. Water retention values of the nanocomposite contact lenses were compared to those of the transparent (untreated) lens and the lens treated through five BI–BO cycles but without NPs at multiple time intervals, to assess not only the steady state value of water retention but also its transient response during water absorption. The final water content values of the transparent, doped without NPs, gold, and silver contact lenses were 45.0, 40.2, 38.4, and 39.6%, respectively. The water retention of the untreated lens was similar to the 46% value reported by Johnson & Johnson for their 1-Day Acuvue TruEye contact lens.^32^ Differences between the water contents of both NCCLs were about 1%; however, the water retention of both nanocomposites was less than that of the untreated contact lens by approximately 5–6%. The diminishing water absorption capacity of the nanocomposites compared to the transparent lens was also noted in their transient water uptake stage. Indeed, the inset of Figure 5c indicates that the initial water uptake of the nanocomposite lenses was at least 10% less than that of untreated lens. However, the behavior of the two nanocomposite contact lenses in retaining water was similar. A possible explanation to this reduction in the water content of the NCCLs is the fact that additional crosslinks may have formed during the breathing-in part of the cycle; these crosslinks would have been between the electron-rich particles of polymeric chains’ functional segments and the gold/silver nanoparticles. The latter could have partially filled up the spaces between the polymeric chains, reduced the effective pore size of the matrix, and diminished the lens’ ability to retain water effectively. Nonetheless, this reduction was not very detrimental, and both the water content and contact angle properties of the nanocomposite contact lenses were within the acceptable range of commercial contact lens products.^33,34^

**Figure 6:**
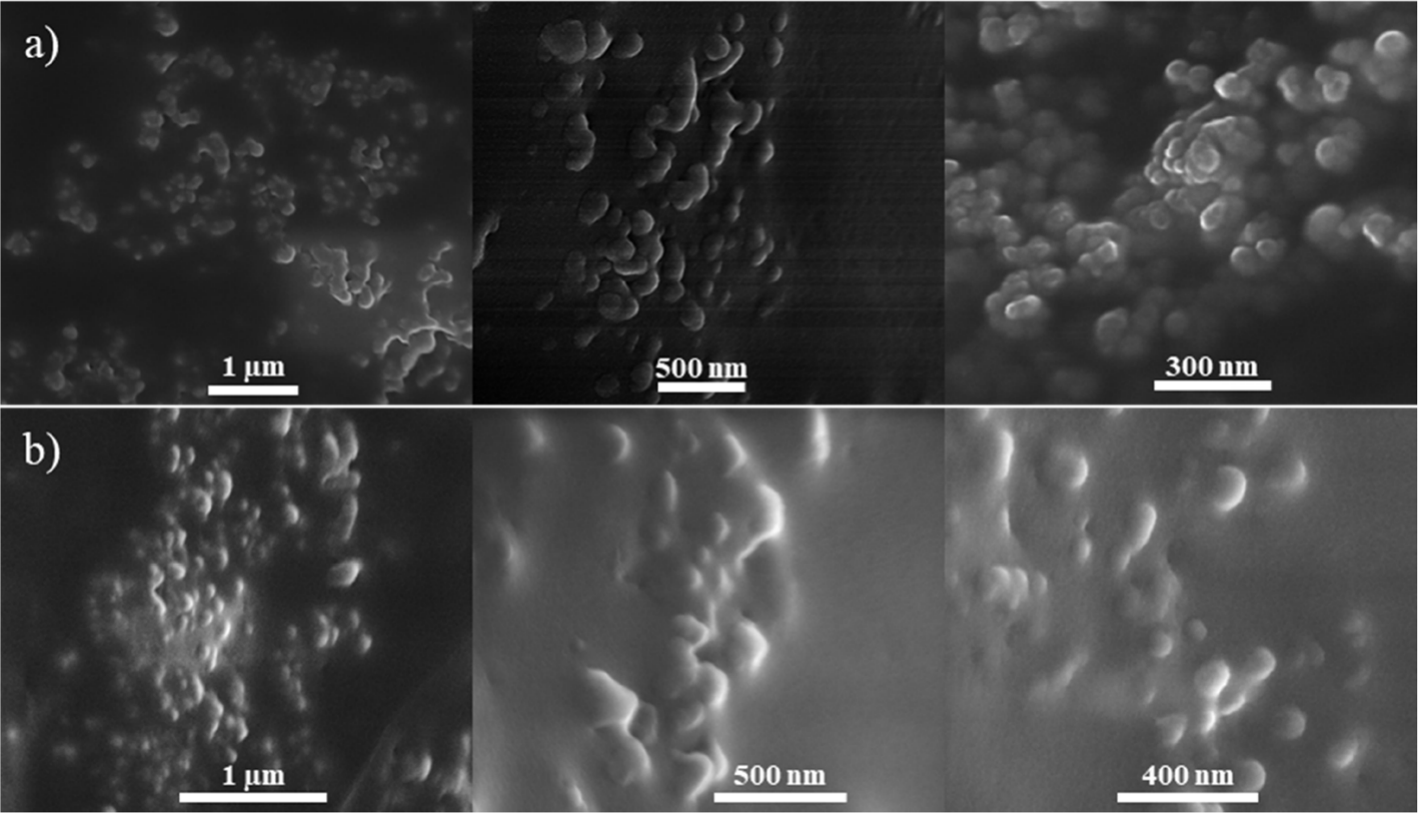
SEM micrographs of the (a) gold and (b) silver nanocomposite contact lens cross-section at different magnifications.

Furthermore, the distribution of both nanoparticles within their contact lenses was examined through SEM imaging of the cross-section, and the resulting micrographs at different scales are shown in Figure 6. Evidently, nanoparticles in both samples were highly aggregated with clear formation of clusters. This was expected as introduction of the nanoparticles into the polymer matrix was not well-ordered. This can also be inferred from Figure 4c,d, which shows that the addition of the nanoparticles to the contact lens through the BI–BO method did not have a consistent change in the lens’ optical properties. Hence, it is no surprise that the nanoparticles were agglomerated, and the latter was noticed in all samples regardless of the BI–BO cycles they have undergone. Nonetheless, the nanoparticles were not all clustered in a specific region within the lens as this would have been evident through the microscopic images, which showed a thorough homogeneous color and no obvious dark spots (Figure 3e,f). Previous studies utilizing the same technique were able to develop highly dispersed nanoparticles and nanowires within the gel.^25,26^ However, they report that coating of the nanoparticles was done to avoid aggregation; authors also regard the homogeneity in NP dispersion to the decrease in the BI–BO cycles or the loading amount of the NPs.^25^ Nanoparticles in this study were purchased and utilized as is, without further modification, and a study done by Salih et al., who used the same set of NPs in situ mixed and polymerized them with a pHEMA hydrogel, also obtained nanocomposites that were highly aggregated and clustered.^21^ It is, thus, worth noting that this method, in itself, did not cause the NP aggregation within the lens.

After characterizing the nanocomposite contact lenses through optical and material tests, their suitability for biomedical and optical applications was assessed by evaluating their efficacy as color filtering wearables and, in particular, as potential blue-light filtering lenses. Recently, detrimental effects of blue light are increasingly being acknowledged, and thus, more light is being shed on the connection between blue-light and eye comfort issues and also on techniques that may combat shortcomings arising from the latter. Further, studies showed that blue light, which has the highest energy in the visible light range, is one of the prominent causes of retinal phototoxicity,^35^ and several animal-based experiments have demonstrated the dangers of long-term exposure to blue light.^36,37^ Melatonin suppression has also been associated with extensive exposure (more than 2 h) to blue light in the evenings. Maximal melatonin suppression was recorded at a light wavelength of 424 nm.^38^ It is also worth noting that not all blue light is harmful as studies have shown that blue light with wavelength ranges of 470–500 nm is crucial for maintaining the normal functionality of several visual functions.^39^ It is reported that high-energy blue light, which causes maximum retinal damage, has a wavelength of 415–455 nm. A good portion of UV radiation is blocked by the cornea, crystalline lens, and other ocular structures; however, the retina is generally exposed to high-energy blue light (400–450 nm), which necessitates the development of blue-light filtering wearables.

**Figure 7:**
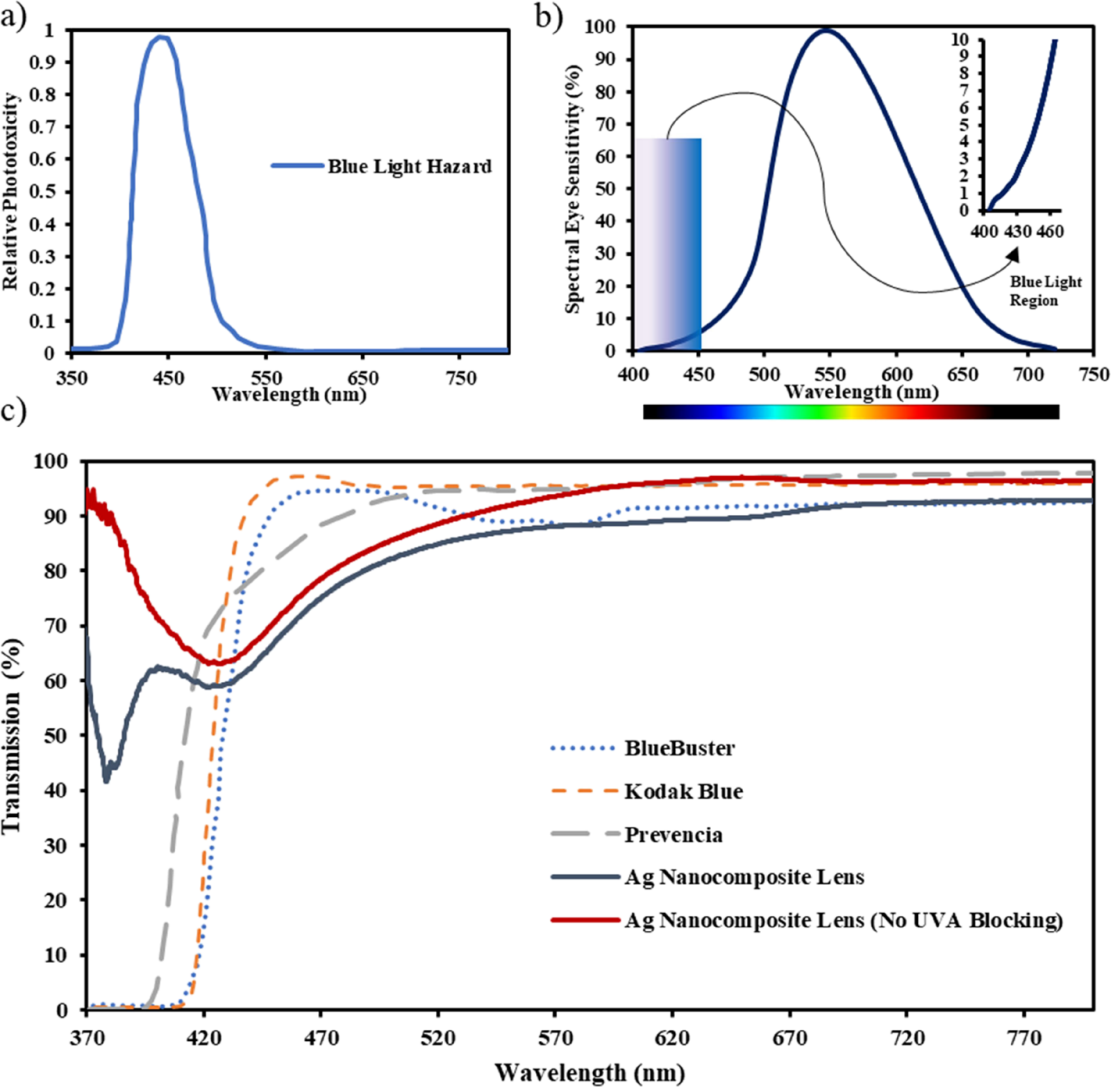
Prominence of blue light and its filtering wearables in ocular discomfort. (a) Retinal phototoxicity spectrum of hazardous blue light, having a peak at 440 nm. Reproduced with permission from ref (39) Copyright 2019 AOSIS. (b) Sensitivity of the eye in the visible light range. Maximum sensitivity occurs at 550 nm, and the inset shows the blue-light part of the region. (c) Transmission spectra of the developed silver nanocomposite contact lenses in comparison to the spectra of commercial blue-light filtering glasses. Commercial products’ spectra were reproduced with permission from ref (39) Copyright 2019 AOSIS.

Hence, to evaluate the filtering efficacy of the silver nanocomposite contact lenses, their transmission spectra are plotted against those of commercial blue-light filtering glasses, which include a product developed by Kodak. The spectra of the silver NCCLs (with and without UVA filter), Kodak Blue, BlueBuster, and Prevencia are shown in Figure 7c. It can be observed that Kodak Blue and BlueBuster block 100% of the light up to 410 nm; both glasses gradually transmit more light, thus reducing their filtering capabilities, from 410 to 450 nm, which is within the high-energy blue-light region. The latter indicates excellent filtering for the UV portion but not the blue-light region. As for Prevencia glasses, it blocks 100% of the light up till 400 nm, after which its transmission steeply increases to 65% at 420 nm. However, unlike the former two glasses, Prevencia glasses have better filtering properties in the high-energy blue-light region, as it steadily blocks an average of 25% of the incoming light from 420–460 nm. Nonetheless, the spectra of the contact lenses were clearly more selective than those of the glasses. The spectra of the silver NCCL without UVA blocking were obtained by using a transparent (untreated) lens as a reference. Light blockage from the silver NCCL at the high-energy blue light, 400–450 nm, was almost constant at 41%, whereas the silver nanocomposite lens without the UV-blocking feature transmitted 72–65% of light at the same range of wavelengths. Both silver nanocomposite contact lenses allowed more than 80% of the incoming light beyond 470 nm.

To sum up, the optical properties of the silver NCCLs were more selective compared to those of commercial wearables, and because their material properties, specifically wettability and water content, are within acceptable ranges compared to other commercial contact lenses, these fabricated NCCLs are well-suited for deployment in this vital application. Utilizing the silver nanocomposite contact lens for blue-light filtering is only one of the potential applications of these developed nanocomposites, as they could also be used as antibacterial contact lenses. Since several studies have reported excellent antibacterial properties of silver nanoparticles,^24,26,40^ the developed contact lenses in this study, which incorporate these NPs, have the potential to combat microbes and bacteria that might adhere to the contact lens. It is worth noting that the BI–BO method would not diminish the antibacterial behavior of the silver nanoparticles as previous studies have shown adequate antibacterial properties of polyacrylamide gel containing silver nanoparticles, which were fabricated through the BI–BO method.^26^ Moreover, since their peak absorption properties are within the range of filtered wavelengths required to facilitate enhanced color distinction for colorblind patients (540–570 nm), the gold NCCLs could be exploited as possible colorblind lenses. Indeed, Salih et al. demonstrated the possibility of deploying such nanocomposite lenses for colorblindness and found that the gold nanocomposites with 40 nm sized NPs displayed the optimum material and optical properties.^21^ Hence, the gold nanocomposite contact lenses, synthesized through the BI–BO method, would probably yield similar if not better properties, as the base contact lens material, herein, is of commercial standard and was not in situ fabricated. Nonetheless, large nanoparticles should be utilized (>40 nm) to filter the desired wavelength range and avoid excessive aggregation.

## 4 Conclusions

Gold and silver nanocomposite contact lenses were successfully synthesized via the unique BI–BO method, for which commercial contact lenses and nanoparticles were utilized. Prior to employing them, the size of the nanoparticles was measured using the captured TEM micrographs, and their transmission spectra were recorded using a spectrophotometer. Upon fabricating the nanocomposite contact lenses, results indicated that the number of BI–BO cycles was directly proportional to the loading amount of NPs within the lens; the latter was confirmed through the recorded transmission spectra. In fact, we showed that by varying the number of cycles, the percentage of light blocked at the surface plasmon could be altered, which is crucial for color filtering applications like color vision deficiency. NP leakage from the fabricated nanocomposite lenses was not observed, and the NCCLs were stable in contact lens storage solution for 1 month. Moreover, the commercially treated contact lenses retained their intrinsic material properties, which was evident from the wettability and water content characterizations. Cross-sectional SEM images of the nanocomposites showed high nanoparticle aggregation; we confirmed that this aggregation was not due to the BI–BO method but rather the NPs themselves, which was outlined in previous studies. The effectiveness of the developed nanocomposites as blue-light filtering lenses was evaluated by comparing their transmission spectra to those of commercial blue-light filtering wearables. The silver nanocomposite lenses had superior optical properties to few of the commercially available glasses; it has not only filtered the range of harmful blue-light wavelengths (400–450) but also transmitted all the light beyond 470 nm, which provides better night vision and color discrimination. Incorporating the NPs into commercial contact lenses, whose properties are well-known and have not been altered, allows for the utilization of these nanocomposites in various optical applications.

## Supporting Information Available

Contact angle setup and measurement procedure, EDX, and additional NP leaching investigation (PDF)
