# Supplementary material for: Syntheses of Gold and Silver Nanocomposite Contact Lenses via Chemical Volumetric Modulation of Hydrogels
Source: ACS Biomater Sci Eng. 2022 Apr 25;8(5):2111–20. doi: 10.1021/acsbiomaterials.2c00174 (PMC9092337; doi:10.1021/acsbiomaterials.2c00174)
Supplement: Supplementary file 1 — ab2c00174_si_001.pdf [file ab2c00174_si_001.pdf]

Supporting Information

The Syntheses of Gold and Silver Nanocomposite Contact Lenses via Chemical Volumetric Modulation of Hydrogels

Ahmed E. Salih,[a,] Mohamed Elsherif,[a] Fahad Alam,[a] Bader Alqattan,[a] Ali K. Yetisen,[b] Haider Butt [a,*]*

[a] Department of Mechanical Engineering, Khalifa University, Abu Dhabi, P.O. Box 127788, UAE

[b] Department of Chemical Engineering, Imperial College London, London SW7 2AZ, UK

*Email: ahmed.salih@ku.ac.ae; haider.butt@ku.ac.ae

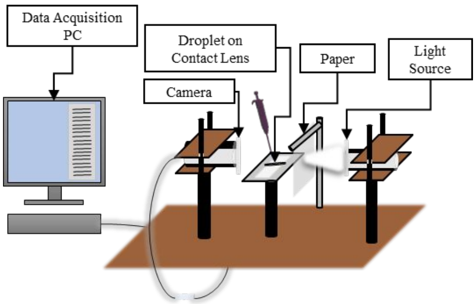

Figure S1: Customized contact angle setup utilized in the study

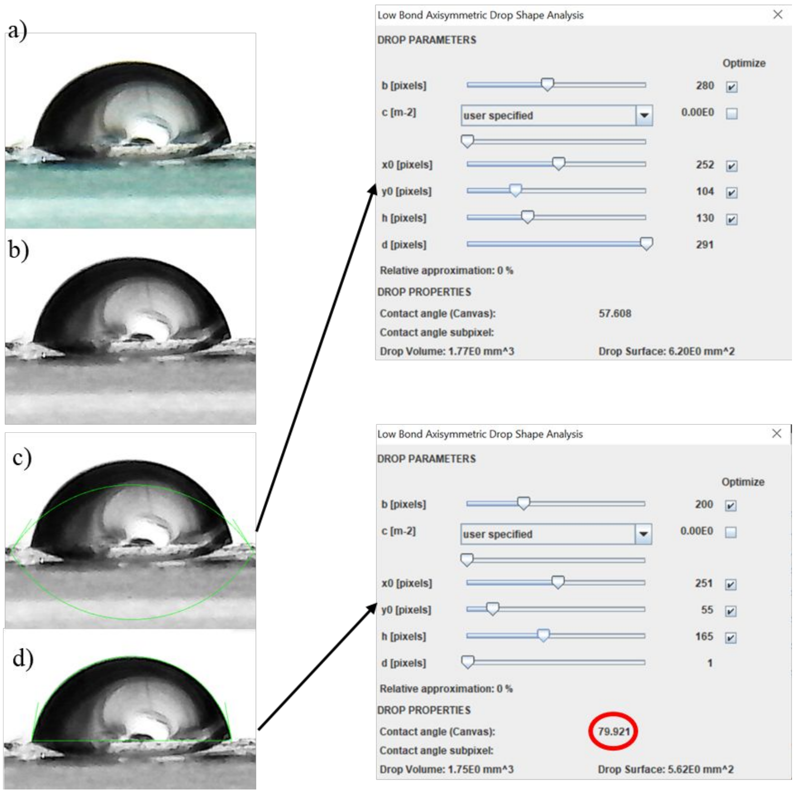

Figure S2: Contact angle measurement steps using ImageJ and "LBADSA" plugin. a) After image is taken as shown in Figure S1, it is imported into the software. b) Image is transformed into greyscale mode. c) "LBADSA" plugin is used to fit the perimeter of the drop. d) Drop parameters are adjusted until the green curve fits the profile of the drop, upon which the contact angle is determined (circled in red).

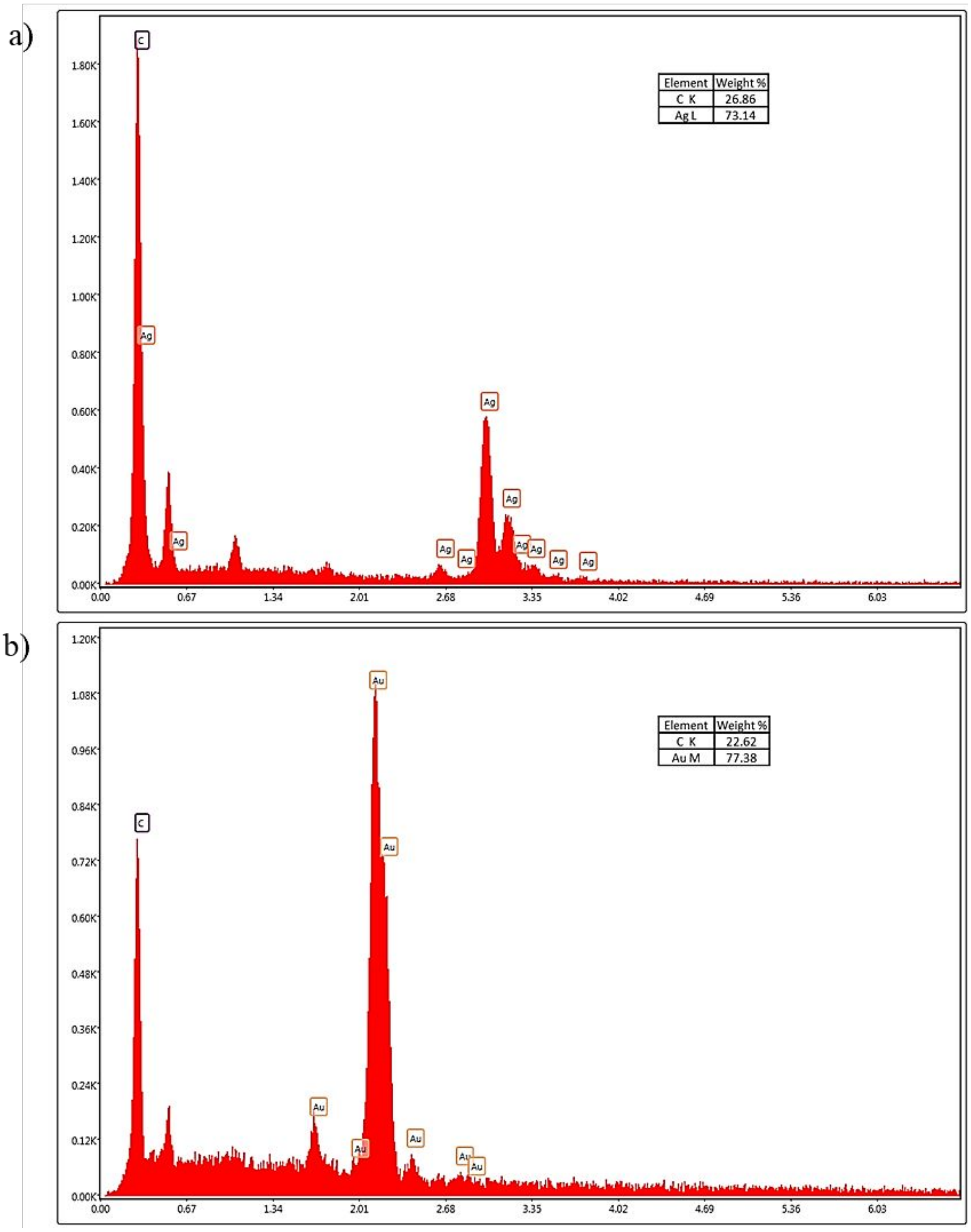

Figure S3: EDX characterization of a) silver and b) gold nanoparticles placed on a carbon tape and analyzed through Nova NanoSEM 650. Inset shows the weight percentage of each chemical element.

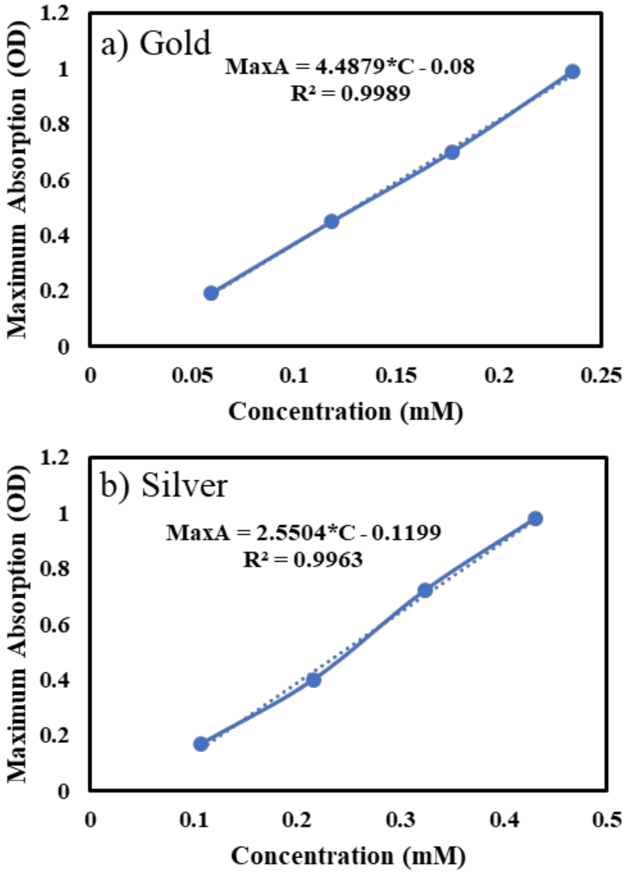

Figure S4: Calibration curve for a) 20 nm gold nanoparticles and b) 40 nm silver nanoparticles used to determine the concentration of the nanoparticles through the measured peak absorption value.

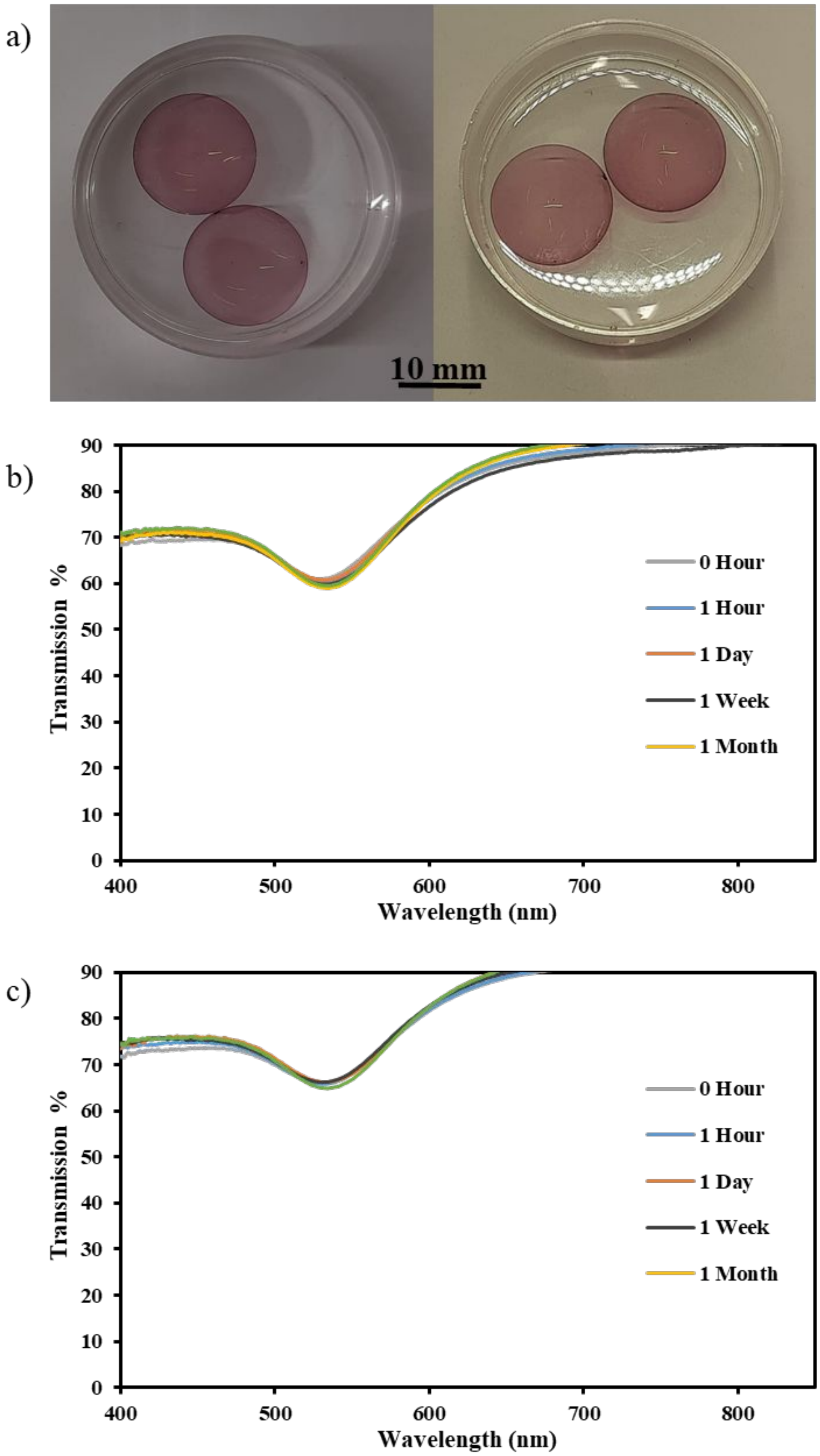

Figure S5: Additional doping experiments done on two lenses using different BI-BO cycles. a) Images of the contact lenses in tear fluid solution. Transmission spectra of lenses undergoing b) 20 and c) 18 cycles over a one-month period while being placed in tear fluid solution.

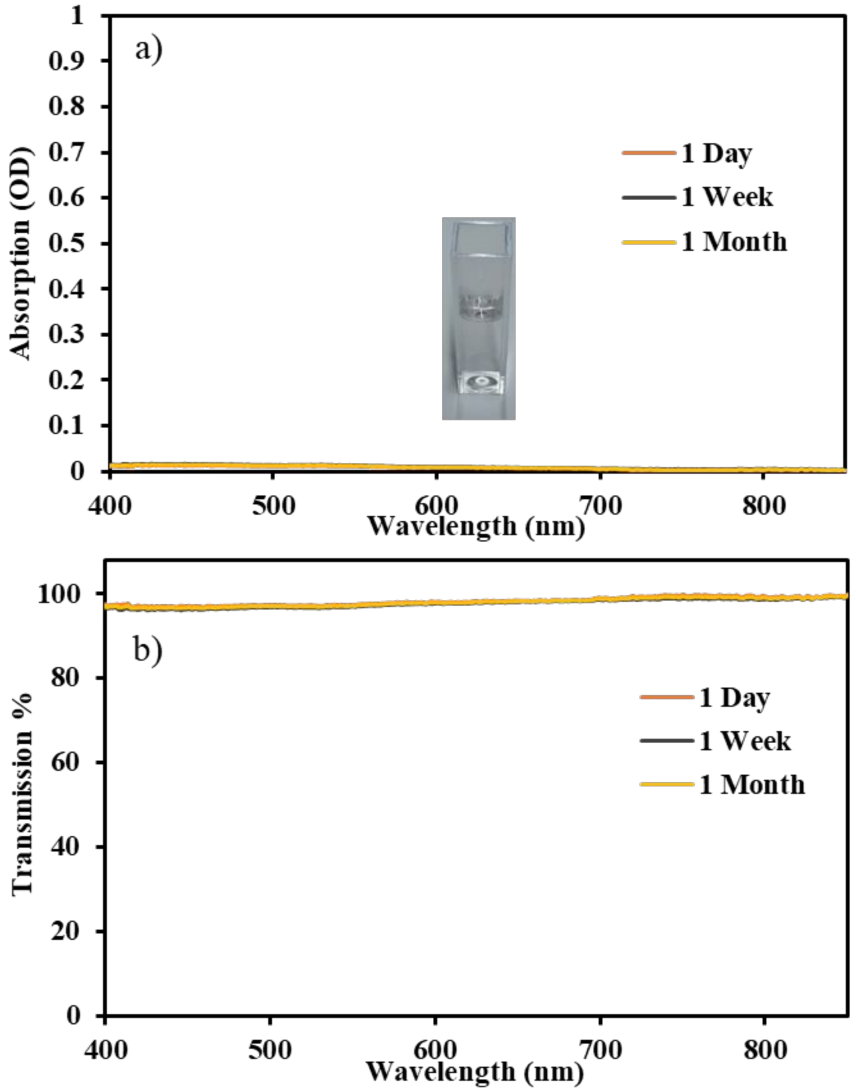

Figure S6: Optical properties of the tear fluid solution in which the nanocomposite contact lenses (Figure S5) were embedded in for a one-month period to examine any possible leaching. a) Absorption and b) Transmission spectra. Inset shows the tear fluid solution after one month.
